# Reliable and Reproducible GABA Measurements Using Automated Spectral Prescription at Ultra-High Field

**DOI:** 10.3389/fnhum.2017.00506

**Published:** 2017-10-25

**Authors:** Yan Li, Wei Bian, Peder Larson, Jason C. Crane, Prasanna Parvathaneni, Srikantan Nagarajan, Sarah J. Nelson

**Affiliations:** ^1^Department of Radiology and Biomedical Imaging, University of California, San Francisco, San Francisco, CA, United States; ^2^Department of Radiology, Stanford University, Palo Alto, CA, United States; ^3^Department of Electrical Engineering, Vanderbilt University, Nashville, TN, United States; ^4^Department of Bioengineering and Therapeutic Sciences, University of California, San Francisco, San Francisco, CA, United States

**Keywords:** GABA, ultra-high field, 7T, magnetic resonance spectroscopy, BASING, spectral editing, reproducibility

## Abstract

**Purpose:** To evaluate spectral acquisition processes important for obtaining reliable and reproducible γ-aminobutyric acid (GABA) signals from volunteers in brain regions that are frequently used for neuroimaging studies [anterior cingulate cortex (ACC), superior temporal gyrus, and caudate] at ultra-high field.

**Methods:** Ten healthy volunteers were studied using a single-voxel Point-RESolved Spectrosocpy (PRESS) sequence with band selective inversion with gradient dephasing pulses (BASING). The editing pulse was designed to be symmetrically placed at 2.0 and 1.4 ppm in the two cycles to reduce the co-editing of macro-molecules (MM). Spectral data were obtained with phase encoding matrix 8 × 8 × 1 and two editing cycles or 1 × 1 × 1 and 64 editing/64 non-editing. The total acquisition time was approximately 4.5 min for each acquisition. An automated MRS prescription method was utilized for the placement of the GABA scan location in 5/10 subjects. Three regions of interest were predefined in the MNI152 space and then registered and transformed to subject space. These volunteers also had repeat scans to examine between-session reproducibility.

**Results:** The placement of editing pulses symmetrically at 1.7 ppm reduced the effect of MM contributions and provided more accurate GABA estimation. Chemical shift misregistration errors caused by classic PRESS localization sequence are more significant at ultra-high field strength. Therefore, a large over-excitation factor was needed to reduce this error. Furthermore, the inefficiency of saturation bands and unspoiled coherence could also interfere with the quality of the data. Reliable recovery of metabolite signals resulted from the implementation of 8 × 8 × 1 phase encoding that successfully removed artifacts and errors, without compromising the total acquisition time. Between successive scans on the same subject, dice overlap ratios of the excited spectral volume between the two scans were in the range of 92–95%. Within subject variability of metabolites between two repeat scans was smaller in the ACC and left superior temporal gyrus when compared to that in the right caudate, with averaged coefficients of variation being 3.6, 6.0, and 16.9%, respectively.

**Conclusion:** This study demonstrated the feasibility of obtaining reliable and reproducible GABA measurements at ultra-high field.

## Introduction

γ-Aminobutyric acid (GABA) is the main inhibitory neurotransmitter in the adult brain, and has been thought to play important roles in many diseases, such as epilepsy (Treiman, 2001) and schizophrenia (Zhang and Reynolds, 2002). The alterations in GABA that are found in these diseases suggest the detection of GABA using proton magnetic resonance spectroscopy (MRS) could provide useful information in understanding the mechanisms of the diseases and monitoring treatment efficacy.

The coupling pattern of the protons in GABA appears as three groups of multiplets (Govindaraju et al., 2000) that partially overlap with resonances from metabolites with higher concentrations, such as creatine (Cr) at 3 ppm. Despite this complex pattern, the detection of GABA is feasible using *in vivo* MRS (Puts and Edden, 2012). The most common acquisition method being applied is an editing sequence, called MEshcher-GArwood Point-RESolved Spectrosocpy (MEGA-PRESS) (Mescher et al., 1998). The difference spectrum between two acquisition cycles, one that inverts the ^3^CH_2_ resonances (edited) and the other without editing, allows the ^2^CH_2_ GABA resonance to be resolved from the CH_3_ resonance of Cr and from a combination of signals from glutamate (Glu), glutamine (Gln), and glutathione (GSH). Spectral data are commonly obtained with a single-voxel acquisition from a volume of 27 cm^3^ in about 10 min at 3T (Mullins et al., 2012).

The increased signal-to-noise ratio (SNR) and improved spectral resolution from ultra-high field (7T) MR scanners that may be beneficial for GABA-edited spectral editing are offset by many complications when using the PRESS sequence, such as chemical shift misregistration errors. The purpose of this study was to evaluate acquisition parameters that are important for obtaining reliable GABA signals from volunteers in the regions that are frequently used for clinical studies at 7T and to examine between-session reproducibility. We used a single-voxel PRESS sequence with band selective inversion with gradient dephasing (BASING) method (Star-Lack et al., 1997) that was similar to MEGA-PRESS with inserting editing pulses into PRESS, but differed in gradient schemes. To examine the reproducibility of such data, we utilized an inhouse-developed automated prescription method, which predefines regions of interest (ROIs) in the MNI152 standard space and then applies them into subject’s space (Bian et al., 2017). The ROIs considered included the anterior cingulate cortex (ACC), superior temporal gyrus, and caudate.

## Materials and Methods

### Study Population

Ten healthy volunteers who had no history of neurologic illness, traumatic brain injury, or cognitive deficiency were recruited into this study. Five of the volunteers were studied to establish the feasibility of the MRS methodology. The other five volunteers (two M and three F, 32 ± 7 years) were used to examine the accuracy and reproducibility of such MR data by repeating the scan twice with a break of 1–5 min outside of the scanner room. Each participant gave written informed consent to undergo the specialized imaging. All the procedures were approved by San Francisco General Hospital Panel Committee at the University of California San Francisco.

### MR Acquisitions

The MR scans were performed using a 32-channel receive-only array with a volume transmit head coil (NOVA Medical, Wilmington, MA, United States) on a GE 7T MR950 scanner (GE Healthcare, Waukesha, WI, United States). Anatomical imaging consisted of a sagittal scout [repetition time (TR)/echo time (TE) = 6/2 ms], 2D fast gradient echo coil sensitivity map (TR/TE = 250/2 ms), and 3D T1-weighted inversion recovery-prepared spoiled gradient echo (IR SPGR) [TR/TE/inversion time (TI) = 6/2/600 ms, matrix size = 256 × 256 × 192, field of view (FOV) = 256 mm × 256 mm × 192 mm, voxel size = 1 mm × 1 mm × 1 mm) images.

**FIGURE 1:**
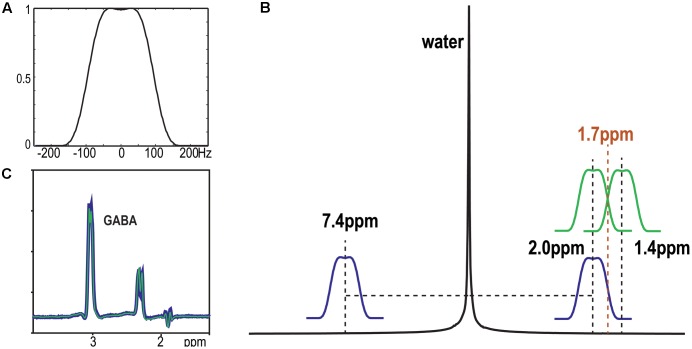
Schematic diagram of the placement of editing pulses **(A)** on the simulated water spectra **(B)** and the difference spectra obtained from a GABA phantom **(C)**. The editing pulses were placed symmetrically to 1.7 ppm (green) or water (blue). No difference was detected on the intensity of GABA between two acquisitions.

**FIGURE 2:**
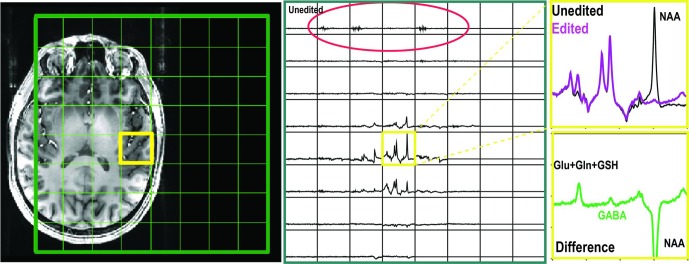
GABA-edited spectra obtained with single voxel volume selection and phase encoding matrix 8 × 8 × 1. The residual signals left by unspoiled coherence were highlighted in red circle. The GABA signal was detected in the selected volume with editing pulse applied symmetric to 1.7 ppm.

GABA-edited MRS was obtained using chemical shift selective (CHESS) water suppression and BASING-PRESS localization with the TE/TR being 68/2000 ms. The editing pulse (**Figure 1A**) was designed to be placed at 2.0 and 1.4 ppm symmetrically to 1.7 ppm (Oz et al., 2006) in the two cycles to reduce the co-editing of macro-molecules (MM) that resonate at 3.0 ppm (Henry et al., 2001). The acquisition applied single voxel volume selection with 8 × 8 × 1 phase encoding steps at a spatial resolution that was defined by the size of the selected volume (**Figure 2**). This is comparable to single-voxel spectroscopy (SVS) acquisitions (1 × 1 × 1) with 64 editing and 64 non-editing repetitions. The total acquisition time was approximately 4.5 min. To reduce the effects of chemical shift misregistration, a 1.9 over-excitation factor was chosen based on a previous study (Li et al., 2015) to excite a volume larger than the required ROI. The volume outside of the ROI was suppressed using very selective suppression (VSS) pulses (Tran et al., 2000). There were six automatically prescribed VSS outer volume suppression bands next to the ROI for the SVS acquisition, while only two VSS bands were placed in the S/I direction for the acquisition with 8 × 8 × 1 phase encoding array. The manufacturer’s linear autoshim procedure was performed before spectral acquisition.

Regions of interest considered were from the ACC, right caudate (RCaud), and left superior temporal gyrus (LSTG). A novel automated spectral positioning of ROIs (Bian et al., 2017) was implemented for this study at 7T. These ROIs were predefined on non-linear T1-weighted average template images in the MNI152 standard space to assess the reproducibility of the acquisition. Linear registration and transformation of locations of ROIs from MNI152 to the subject’s space were applied automatically using an inhouse-developed software. The voxel size was fixed for each ROI at 2 cm × 2 cm × 2 cm, 2 cm × 2 cm × 2 cm, and 2.5 cm × 2.5 cm × 2 cm for the ACC, RCaud, and LSTG, respectively. The parameters defining the position of the MRS prescription were then passed directly to the spectral sequence. The total time for this procedure was about 1–2 min for each ROI.

### MR Post-Processing

The T1-weighted IR SPGR images were processed with N4 bias correction (Tustison et al., 2010), brain extraction, and Atropos probabilistic tissue segmentation to generate masks of gray matter, white matter, and cerebral spinal fluid (Avants et al., 2011). The mask of the caudate was segmented using the Harvard-Oxford subcortical structural atlas (Frazier et al., 2005) from the FSL software package. The segmented masks were then aligned to the orientation of the spectra data, followed by calculating the percentage of these components within each spectral voxel.

The GABA-edited BASING-PRESS data were processed and quantified using similar methods to those described in a previously published consensus paper (Mullins et al., 2012). The single voxel data were processed with phase and frequency corrections individually for each coil, and then combined by weighting with coil sensitivities (Li et al., 2015), while the spectral data acquired with 8 × 8 × 1 phase encoding steps were first fast Fourier transformed in the k-space domain with half voxel in-plane shift. The difference spectra, the subtraction of edited from non-edited spectra, were quantified by LCModel (Provencher, 1993) using an *in vitro* basis-set of individual metabolites, consisting of *N*-acetylaspartate (NAA), Glu, Gln, GABA, GSH, and *N*-acetylaspartylglutamate.

### Data Analysis

Within-subject analysis of re-positioning the excited volume was evaluated by the dice overlap ratio (Dice, 1945) for each ROI. The T1-weighted IR SPGR images from the second section were rigidly co-registered to the first images using FSL FLIRT (FMRIB’s Linear Image Registration Tool) (Jenkinson et al., 2002), and then the volume of the ROI at each time point and the portion that overlapped were calculated (Hancu et al., 2005; Bian et al., 2017). The total shift of the center of volume was calculated by the square root of the sum of the square of the shift in three orthogonal dimensions between the two scans.

Cramer-Rao lower bounds (CRLBs) are used as indicators for reliability of LCModel quantification (Cavassila et al., 2000). Metabolites with CRLB lower than 20% were included in the analysis. The coefficients of variation (CVs) were calculated for each metabolic profile using the standard deviation of the two measurements divided by the mean for test–retest reproducibility. Descriptive statistics of segmented tissues components, prescription PRESS volume, and metabolic profiles was calculated for between-subject analyses. A paired *t*-test was utilized to assess the differences in metabolite ratios between-session. *P*-values of 0.05 or smaller were considered to be significant.

## Results

### Reduced Effects of MM

**FIGURE 3:**
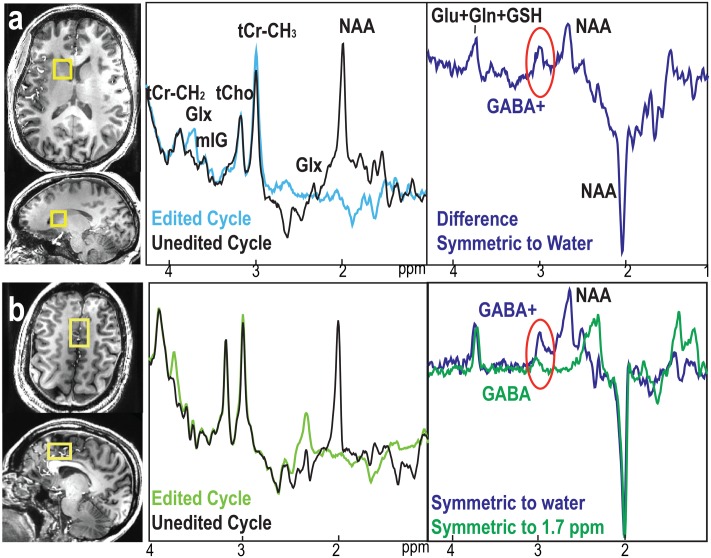
Examples of GABA-edited BASING-PRESS from volunteers. **(a)** Edited, non-edited, and difference spectra using editing pulses placed symmetrically to water from RCaud (right caudate). **(b)** Edited, non-edited, and difference spectra using editing pulses placed symmetrically to 1.7 ppm (green) compared to the difference spectra using editing pulse symmetrically to water (blue) from the anterior cingulate cortex (ACC). GABA+ = GABA + MM.

The placements of editing pulses (**Figure 1B**) symmetrically to 1.7 ppm and water are shown in **Figure 1A**. The difference spectra from the phantom with GABA only showed no difference in detecting GABA between these two acquisitions (**Figure 1C**). For the *in vivo* dataset, the MM resonating at 3.0 ppm couple to spins at 1.7 ppm and were co-edited in the editing cycles. This results in an overestimation of GABA when editing pulse is placed symmetrically to water and an index known as GABA+ (GABA+MM) is commonly used (**Figure 3a**). The placement of editing pulses at 2.0 and 1.4 ppm can remove this effect. **Figure 3b** illustrates an example of the difference between GABA+ and GABA from the ACC. After quantification using LCModel, the level of GABA+ and GABA relative to NAA was 0.58 vs. 0.21, respectively. In this case, the ratio of GABA/NAA was over-estimated by 1.76 times higher when MM was co-edited.

### Elimination of Unwanted Signals

**FIGURE 4:**
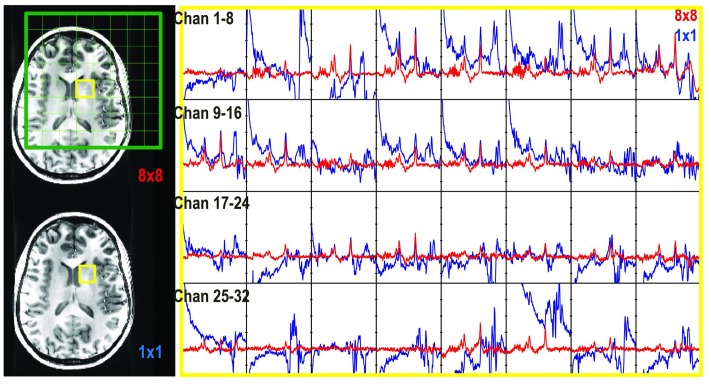
Unedited spectra obtained with 1 × 1 × 1 (64 averages, blue) and 8 × 8 × 1 (red) phase encoding steps. The spectra in the selected voxel were plotted for each individual channel (channels 1–32).

Chemical shift misregistration that is caused by the PRESS RF pulses was significant at 7T (**Figure 2**). An over-excitation factor of 1.9 was utilized to ensure all metabolites were excited in the voxel of interest. **Figure 4** also shows an example of unedited spectra in each individual channel that was acquired using both 1 × 1 × 1 and 8 × 8 × 1 phase encoding. The phase encoding steps successfully removed the residual signals left by the saturation bands and unspoiled coherence that could interfere with the quality of data.

### Reproducibility of the Voxel Placement

**FIGURE 5:**
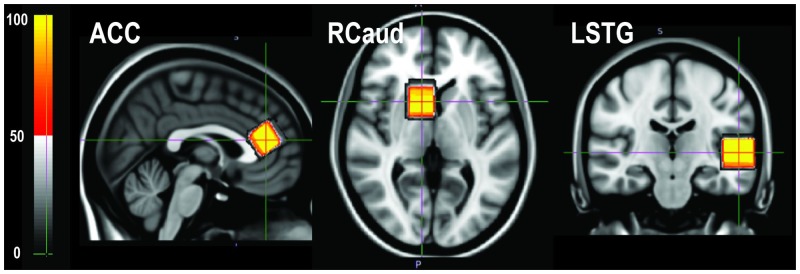
The voxel position from 10 volunteer scans transformed back to the MNI152 standard space and overlaid on the template image. The color bar indicates the percentage of overlapping.

The within-subject dice overlap ratios of the excited spectral volume between the two scans were 0.92 ± 0.04, 0.93 ± 0.04, and 0.95 ± 0.02 for ACC, RCaud, and LSTG, respectively. The total shifts on the center of the PRESS box were 1.05 ± 0.61, 0.91 ± 0.47, and 0.27 ± 0.27 mm for ACC, RCaud, and LSTG, respectively. **Figure 5** shows the location of the PRESS boxes from 10 volunteer scans overlaid on the T1-weighted average template images in the MNI152 standard space. The variation on the inclusion of the caudate was 3.3 ± 3.8% (CV) with the mean percentage of caudate within the PRESS box being 26.1%. The differences in between-subject segmented tissue components for each ROI are illustrated in **Table 1**.

**Table 1:** Mean and standard deviation of the percent of tissue components within spectral voxel.

	GM	WM	CSF
ACC	56.1 ± 4.9	23.1 ± 4.4	20.8 ± 5.4
RCaud	39.1 ± 3.0	42.2 ± 6.4	18.7 ± 4.6
LSTG	41.3 ± 5.4	32.5 ± 5.4	25.9 ± 9.0

*ACC, anterior cingulate cortex; RCaud, right caudate; LSTG, left superior temporal gyrus; GM, gray matter; WM, white matter; CSF, cerebrospinal fluid.*

### Reproducibility of Metabolic Levels

From the non-edited spectra, the linewidths of Cho, Cr, and NAA within the ACC were 9.7 ± 3.9, 9.6 ± 4.5, and 19.1 ± 3.1 Hz, respectively; in the RCaud they were 24.2 ± 8.0, 24.1 ± 4.4, and 27.3 ± 3.9 Hz, respectively; and in the LSTG they were 5.5 ± 4.0, 7.3 ± 4.4, and 20.4 ± 5.4 Hz, respectively. The metabolite ratios, CRLB, and CVs in the ACC, RCaud, and LSTG for GABA measurements are summarized in **Table 2**. The averaged CVs for Cho/NAA from the non-edited cycle were 0.032, 0.100, and 0.101 for ACC, RCaud, and LSTG, respectively. No statistically significant difference was found between-session for GABA/NAA and Cho/NAA (*p* > 0.05, *N* = 15).

**Table 2:** Cramer-Rao lower bound (CRLB) (top rows), GABA/NAA ratio (middle rows), and CV (bottom rows) from five volunteers for each ROI.

	Subject #1		Subject #2		Subject #3		Subject #4		Subject #5	
	Scan 1	Scan 2	Scan 1	Scan 2	Scan 1	Scan 2	Scan 1	Scan 2	Scan 1	Scan 2
ACC	7%	7%	10%	13%	10%	9%	10%	7%	7%	7%
	0.106	0.100	0.082	0.082	0.098	0.102	0.099	0.108	0.085	0.081
	4.1%		0.0%		4.5%		6.1%		3.4%	
RCaud	15%	16%	9%	15%	17%	12%	21%	12%	14%	8%
	0.081	0.105	0.156	0.119	0.127	0.110	0.098	0.140	0.127	0.151
	18.2%		19.0%		10.1%		25.0%		12.2%	
LSTG	8%	6%	8%	6%	7%	7%	10%	13%	6%	7%
	0.128	0.144	0.091	0.099	0.083	0.075	0.108	0.103	0.104	0.112
	8.3%		6.0%		7.2%		3.4%		5.2%	

*CRLB, Cramer-Rao lower bound; ACC, anterior cingulate cortex; RCaud, right caudate; LSTG, left superior temporal gyrus.*

## Discussion

In this study, we implemented and evaluated an automated MRS method for obtaining reproducible GABA signals from the healthy brain. The results demonstrated that it is important to use specific acquisition parameters to obtain reliable GABA-edited BASING-PRESS MRS at ultra-high field strengths. An automated prescription of the volume of interest was employed to facilitate the acquisition process and to ensure similar anatomic location within- and across subjects. Our results on the test–retest agreement of voxel positioning and metabolic profiles are promising in terms of being able to use this method for evaluating and managing patients in a clinical setting.

Spectral editing methods apply selective inversion pulses based on prior knowledge of the coupling patterns of the metabolite of interest and overlapping resonances. The MEGA-PRESS sequence has been shown to provide reproducible within- and between-session GABA measurements at 3T (Brix et al., 2017; Yasen et al., 2017). However, this method also co-edits signals from MM (Behar and Ogino, 1993; Behar et al., 1994), resulting in over-estimation of GABA that needs to be accounted for (Bhattacharyya, 2014). It has been reported in the literature that MM contamination on GABA measurements was 46% at 3T (Near et al., 2011) and 17% at 7T (Terpstra et al., 2002). The levels of MM were also found to be different between gray and white matter (McLean and Barker, 2006), which could cause inaccuracies in GABA measurements. Methods to reduce this effect include acquiring additional metabolite-null spectrum using inversion recovery preparation (Behar et al., 1994; Terpstra et al., 2002), a relatively long TE (e.g., 80 ms) (Edden et al., 2012), or applying editing pulses symmetrically at 1.7 ppm (Henry et al., 2001). The latter method requires the editing pulses to have a relatively narrow inversion profile and short duration. The improved spectra resolution of 7T can make it less challenging to design such editing pulses.

The use of ultra-high field strength has been shown to provide a considerable advantage over conventional 1.5T and 3T scanners for the acquisition of MRS. Higher SNR, better spectral resolution, and more accurate quantification have been reported at 7T for SVS and magnetic resonance spectroscopic imaging (Otazo et al., 2006; Mekle et al., 2009; Tkac et al., 2009; Lupo et al., 2011; Nelson et al., 2013). These improved capabilities allow for the detection of GABA, Glu, and Gln using short TE MRS without editing (Henning et al., 2009; Tkac et al., 2009; Li et al., 2015). A recent study reported that MEGA-PRESS provided modest reproducibility in the regions of ACC and dorsolateral prefrontal cortex at 7T (CV: 13.6%, 13.4%) as well as short TE STEAM (CV: 3.5%, 16.2%) (Wijtenburg et al., 2013). These results encourage the application of spectral editing MRS sequence for volunteers and patients to obtain reliable neurochemical profiles at ultra-high field strengths.

In this study, we applied similar editing sequences in conjunction with BASING rf pulses for editing GABA at 7T. Although PRESS has significant chemical misregistration at 7T, a 1.9 over-excitation factor was applied to reduce this error (Li et al., 2015). This relies upon outer volume suppression pulses to suppress signals arising from beyond the ROI. We found that using 8 × 8 × 1 phase encoding steps was able to eliminate unwanted signals from outside of the selected volume of interest after using crusher gradients (**Figure 2**). However, the point spread function for a CSI acquisition can lead to intervoxel signal leakage, with neighboring signal contributing to the voxel. In contrast to the most commonly available PRESS localization, an alternative is to use LASER (Garwood and DelaBarre, 2001) or semi-LASER (sLASER) methods (Scheenen et al., 2008). The sLASER scheme has been integrated with GABA editing, MEGA (Andreychenko et al., 2012), or J-difference editing (Prinsen et al., 2017). The latter method provided a CV of 9.5 ± 7.0% for GABA in a region of 3 cm × 3 cm × 3 cm with 128 editing-on and 128 editing-off repetitions.

Regional variances on the concentrations of GABA, Glu, and Gln were reported in the ACC. Pregenual ACC was found to have significantly higher GABA, Glu, and Gln concentrations than those in the anterior mid-cingulate cortex (Dou et al., 2013). For the regions without obvious landmarks, such as the superior temporal gyrus, easy, fast, and reproducible voxel positioning is important when investigating variation across subjects or evaluating temporal changes in metabolic profiles. Our automated spectral prescription method provided great accuracy of repositioning the excited volume with dice overlap ratios of 92–95% in this study. For a larger predefined volume, such as 8 cm × 10 cm × 5 cm, even better overlap (97.8%) was achieved (Bian et al., 2017). Tissue components based on brain segmentation showed a relatively small variation within- and between-subject. As a result, no correction for tissue water and CSF contributions was applied for metabolite quantification in this study.

One concern in obtaining reliable spectroscopy data at 7T is due to increased inhomogeneity. In this study, the commercially available GE linear shimming method was used to achieve B0 homogeneity at 7T and provided good spectral quality in the ACC and LSTG, with a mean value of 9.6 and 7.3 Hz for Cr, respectively. The caudate was more difficult to shim and had a large linewidth for Cr (24.1 Hz). Improved shimming methods, such as the use of second-order shim and FASTERMAP (Shen et al., 1997), will be needed to improve spectra quality for future studies. This may also explain why the reproducibility of GABA measurement in the RCaud was worse compared to those in the ACC and LSTG. Increasing the number of repetitions could improve within-subject reproducibility (Brix et al., 2017). The averaged CVs for GABA/NAA in the ACC and LSTG (3.6 and 6.0%) were similar to those observed in previously published data (Prinsen et al., 2017; Yasen et al., 2017).

This study established the feasibility of obtaining reliable and reproducible GABA measurements in three different ROIs from healthy volunteers at ultra-high field strengths. The symmetric placement of editing pulse was inserted into the sequence to reduce the effect of GABA overestimation. This method is also vulnerable to field drift between editing-on and editing-off cycles. Performing cardiac triggering was suggested to improve phase and frequency stability for spectra editing acquisitions (Andreychenko et al., 2012). In this study, we evaluated test–retest agreement on GABA measurement using the most common localization method. Although phase encoding steps eliminated unwanted signals without compromising on the total acquisition time or using in-plane saturation bands, it would add extra time when acquiring additional unsuppressed water data. Since the purpose of the study was to evaluate different acquisition parameters and we were successful in prescribing very similar voxel locations, we did not apply tissue corrections and water scaling when reporting metabolite levels.

## Conclusion

This study evaluated acquisition processes that are important for obtaining reliable GABA signals from volunteers in the regions that are frequently used for clinical studies at ultra-high field strengths using an automated spectral prescription. This method provided highly reliable and accurately localized GABA signals with a relatively short acquisition time (4.5 min), enabled GABA detection in small voxels (8 cm^3^) and at short TE (68 ms) without GABA overestimation.

## Author Contributions

PL designed the editing pulse, WB and JC implemented automatic MRS prescription software on the scanner, and PP performed image segmentation. YL carried out study design, sequence implementation, data acquisition, analysis, and wrote the first draft of the manuscript. SN and SJN oversaw the project. All co-authors contributed to interpretation of results and have approved the manuscript.

## Conflict of Interest Statement

The authors declare that the research was conducted in the absence of any commercial or financial relationships that could be construed as a potential conflict of interest. GE Healthcare provides a research grant to fund part of this study.
